# Potential antitumor and anti-inflammatory activities of an extracellular polymeric substance (EPS) from *Bacillus subtilis* isolated from a housefly

**DOI:** 10.1038/s41598-022-05143-9

**Published:** 2022-01-26

**Authors:** Lingxiu Zhang, Huilan Yi

**Affiliations:** 1 School of Life Science, Shanxi University, No. 92 Wucheng Road, Taiyuan, 030006 China; 2 College of Environment and Resource Sciences, Shanxi University, Taiyuan, 030006 China; 3 Department of Biology, Xinzhou Teachers University, Xinzhou, 034000 China

**Keywords:** Microbiology, Medical research, Molecular medicine, Drug discovery, Drug screening, Immunology, Immunotherapy

## Abstract

*Bacillus subtilis*, a probiotic, has been applied in the medical, food, and feed industries among others. However, the mechanisms of its benefits to hosts are not yet fully understood. Here the characterization and bioactivities of an extracellular polymeric substance (EPS) from *Bacillus subtilis* were investigated to reveal its partial mechanisms and provide the theoretical basics for further development and utilization of *Bacillus subtilis*. In this study, the novel strain *Bacillus subtilis* xztubd1 (GenBank: MG458322.1) was isolated from a housefly’s body, identified according to phenotypical and genotypical analyses, and found to produce large amounts of an EPS. Through ultraviolet spectroscopy and Fourier transform infrared spectroscopy (FTIR spectroscopy), the EPS was found to contain a variety of chemical functional groups, such as O–H groups, C=C, C=O, CH_3_, C–O–H and C–O–C bonds, and alpha-type pyranose. Furthermore, the in vitro antioxidant activity of the EPS on DPPH radicals at a concentration of 90 μg/ml was 62%; on the superoxide radical at a concentration of 90 μg/ml, this value was 75%; and on hydroxyl radicals at a concentration of 90 μg/ml, the activity was 54%. EPS also enhanced significantly phagocytosis, lysozyme activity in macrophages, IL-2 content in mice and inhibited dramatically the growth of HeLa cells. These results showed that the EPS with reductive groups have the strong capacity to scavenge reactive oxygen species (ROS), reinforce the immune system and inhibit the growth of cancer cell, which helps theirs hosts defence against many diseases, including inflammation and cancer. The EPS from *Bacillus subtilis* has the potential to be an anticancer and anti-inflammatory drug candidate in the pharmaceutical industries, which provide scientific evidence for the development and utilization of probiotic-derived medicines.

## Introduction

The exploitation of microbial resources always has great research value and bright prospects with the ability to produce various bioactive substances^1,2^. Intestinal probiotics provide health benefits to humans and other animals by decreasing pathogenic microorganisms, strengthening the immune system, protecting against oxidative damage, reducing carcinogenesis and protecting the intestinal barrier through the secretion of beneficial substances^3–6^. Interestingly, probiotics can also prevent and aid in the treatment of the diseases caused by SARS-CoV-2^7–9^. The effects of probiotics involve live microorganisms, microbial DNA, soluble proteins, cell wall components and metabolites^10,11^. However, the mechanisms of their benefits to hosts are not yet fully understood.

*B. subtilis* has been demonstrated to be a probiotic that provides many advantages to hosts, including forming biofilms, secreting antimicrobials, and preventing inflammatory diseases by modulating the innate immune response and T cell-mediated immune responses^12,13^. Exopolysaccharides (EPSs) are important high-molecular-weight homo- or heterobiopolymers secreted by microorganisms that have become a very hot topic due to their many biological activities^14–16^. Recently, it was found that exopolysaccharides from *Bacillus subtilis* had antioxidant activity, anticoagulant efficacy, and antitumour properties^17–20^. However, the novel probiotic still needs to be isolated and identified to meet a variety of requirements for human health. New species of *Bacillus subtilis* not only provides new species resources but also produces more microbiological products that may possess potential application value. Furthermore, new structural and functional EPSs may further reveal their beneficial mechanism.

Reactive oxygen species (ROS) mainly produced by mitochondria may lead to permanent cell damage by oxidation of proteins, phospholipids, and DNA. The overgeneration of ROS disrupts the tissues and organs and cause death of the organism. Consequently, ROS have been associated with many pathological conditions as important regulators of physiological cell signalling, such as aging, tumorigenesis and inflammation. Ascorbic acid is commonly used as anti-oxidants to remove excess ROS, which is only used as adjuvant therapy for diseases because of its single function^21^.

People, especially children and the old, with low immune function was easily infected and increased susceptibility to cancer because of inability to remove pathogenic bacteria and mutant cells in time. Treatment methods of the immunodeficiency include increasing the nutrient and eating immune-boosting foods.

The death rate of cancer is has always been gradually declining, which attributed to advances in treatments. Surgery, radiotherapy, medical treatments (chemotherapy, targeted therapies, immunotherapy) were adopt in the treatment of cancer among which developing new cancer drugs is the most common and basic treatment.

In this study, the novel probiotic *Bacillus subtilis* xztubd1 was isolated from a housefly, and the structure and function of its EPS were evaluated including chemical functional groups, antioxidant activity, phagocytosis, lysozyme activity in macrophages, IL-2 content and the suppression rate of HeLa cells. Our results reveal a potential mechanism for how EPS offer benefits to the hosts and may have the potential to be used to prevent cancer and inflammation. Our findings provide scientific evidence for the development and utilization of the EPS from *Bacillus subtilis*.

## Results

### Identification of the microbial strain

**Figure 1:**
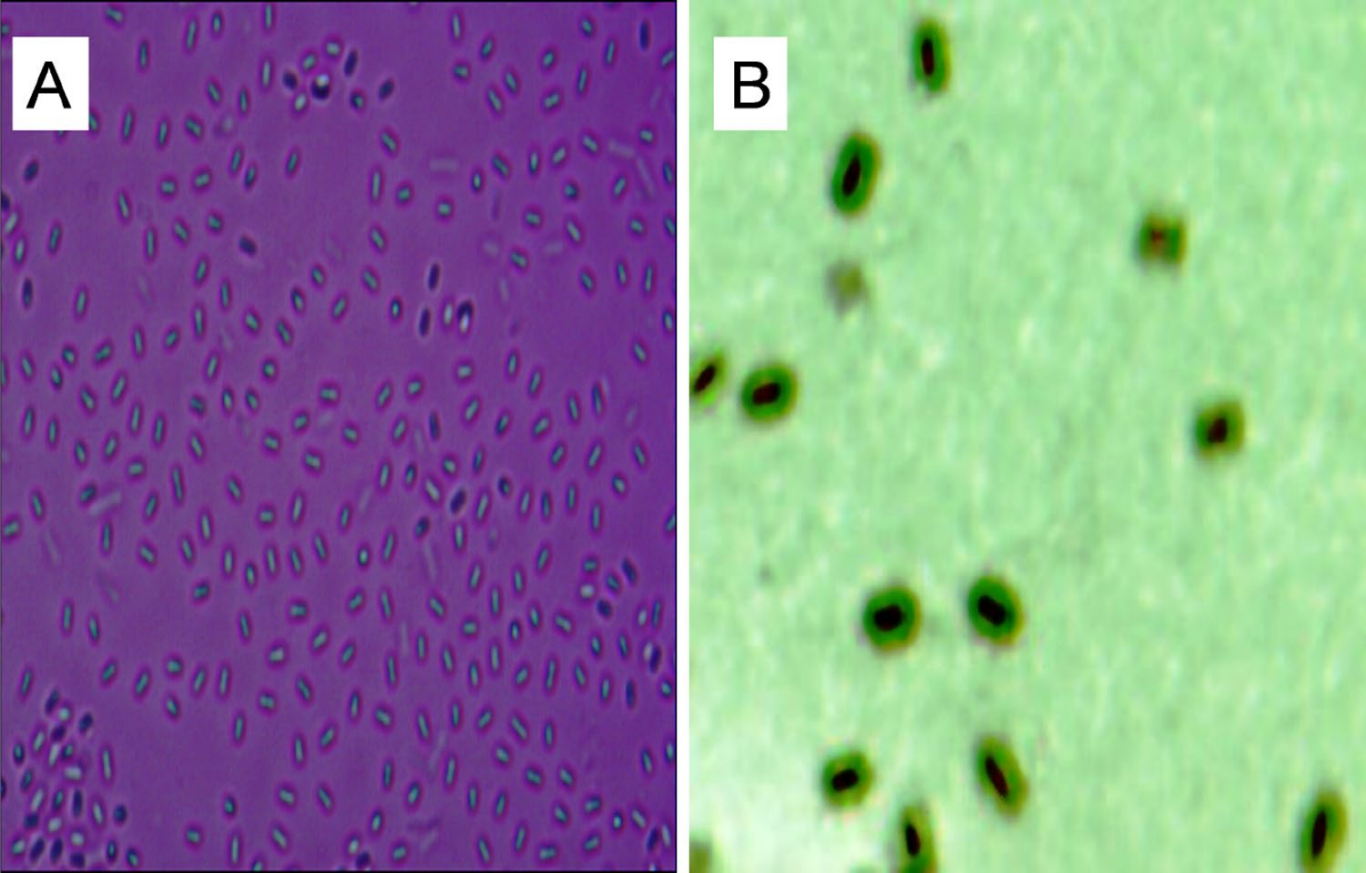
The microgram of *Bacillus subtilis* xztubd1. Magnification: 100 × (**A**) and 400 × (**B**).

**Figure 2:**
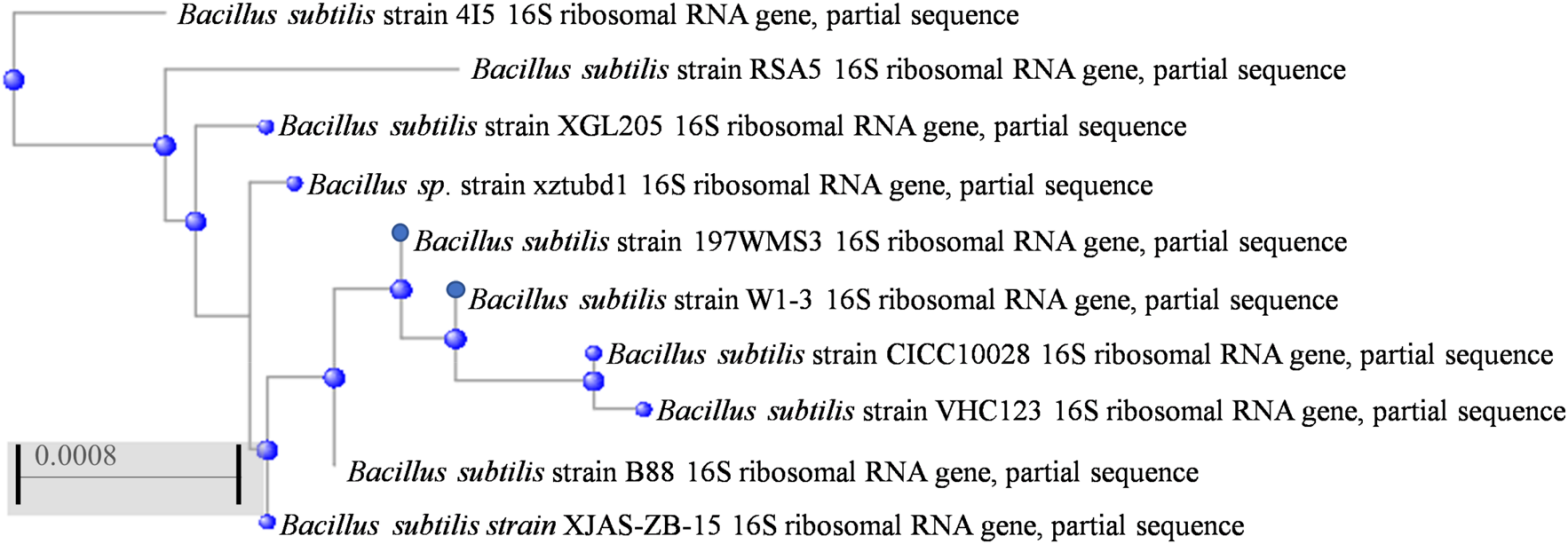
Phylogenetic tree of *Bacillus subtilis* xztubd1.

The bacteria isolated from a housefly were found to secrete a large amount of EPS to further study. By microscopic observation, the bacteria were rod-shaped, characterized by a tough endospore and had a loose layer of mucous material named the capsule (Fig. 1). Moreover, 16S rDNA gene sequencing analysis showed that the species was *Bacillus subtilis*, as it was highly related to other *Bacillus subtilis* strains at 99% similarity (Fig. 2). Therefore, according to the morphological characterization and 16S rDNA gene sequencing analysis, the bacterium was identified as *Bacillus subtilis*. The 16S rDNA gene sequences of *Bacillus subtilis* xztubd1 were deposited in GenBank under the accession numbers MG458322.1.

### UV–vis and FTIR characterization

**Figure 3:**
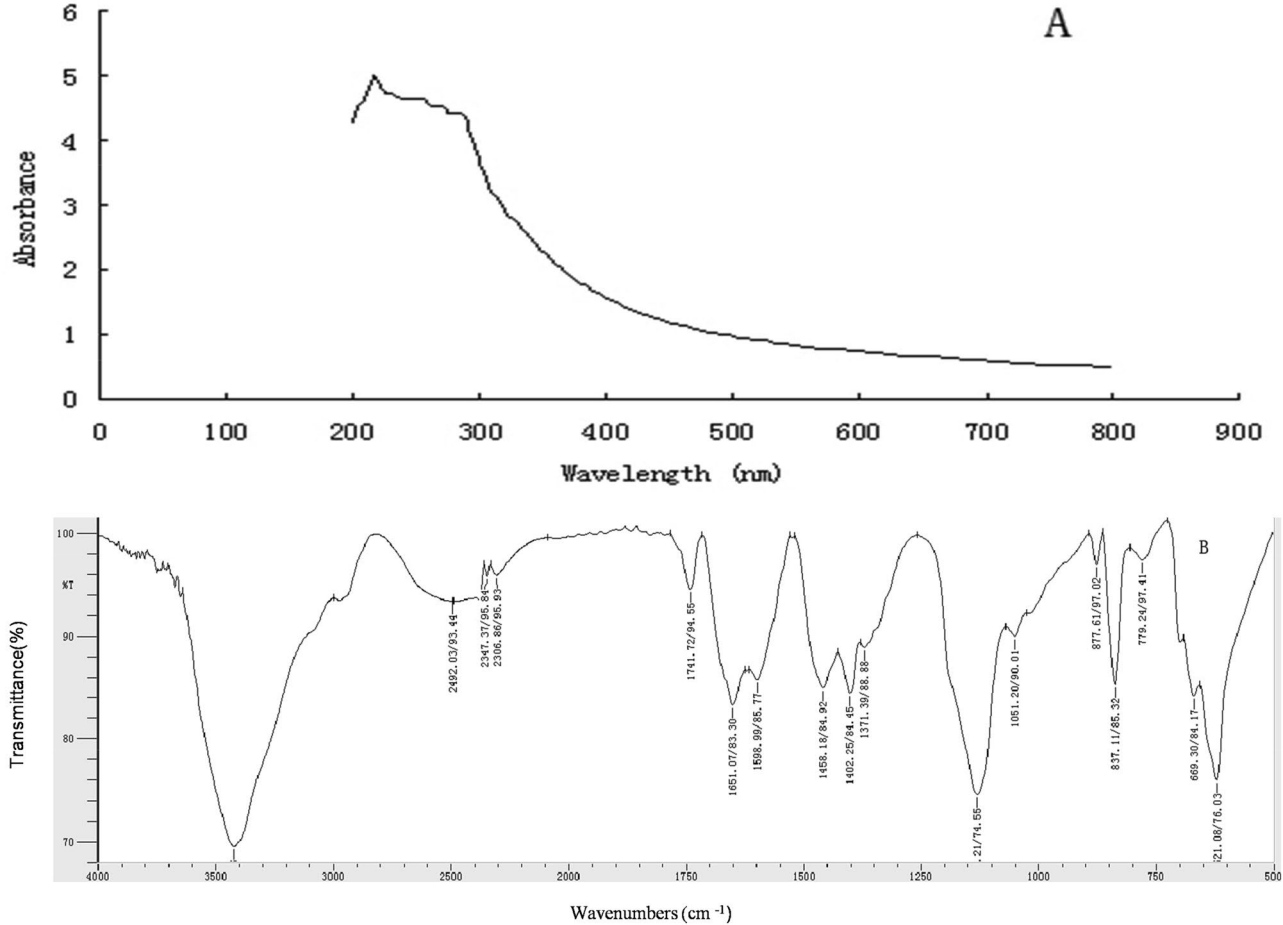
UV–vis and FTIR spectra of the EPS from *Bacillus subtilis* xztubd1.

UV–vis and FTIR spectroscopies are techniques that measure the absorption of radiation, which determines particular compositions and structures of substances in a sample. As shown in Fig. 3, the UV–vis spectrum of EPS showed an obvious absorbance peak from 200–3300 nm, which was caused by the n–σ* and/or π–π* transition of a conjugated diene or an α,β-unsaturated aldehyde and/or ketone, indicating that EPS contained one or more functional groups, such as carboxy, carbonyl, or ester groups. According to the FTIR characterization, the functional groups and glycosidic bonds of EPS corresponding to the absorption peaks were speculated. Absorption peaks at approximately 3421 cm^−1^ and 2492 cm^−1^ were caused by stretching vibrations of OH and C=C, respectively. Bands at approximately 2347 cm^−1^ and 2306 cm^−1^ were derived from the bending vibration of CO_2_. The stretching band at approximately 1741 cm^-1^ was due to the stretching vibration of C=O bonds. Characteristic bands at 1651/1598 cm^−1^ corresponded to the stretching vibration of C=C. The bands at 1458 cm^−1^ and 1402 cm^−1^ were attributed to the stretching vibration of CH_3_. Broad and intense absorption bands due to C–O stretching vibrations were observed at 1132 cm^–1^ and 1051 cm^−1^, indicating the presence of sugar rings consisting of C–O–H and C–O–C. The band at 837 cm^−1^ indicated the presence of α-pyranose.

It has been considered that the molecular functions and mechanism of action are related to the structure of a compound. To confirm the mechanism of enhanced immunity, we examined the functional groups of EPS by UV–vis and FTIR spectroscopies. These findings provide a new theoretical basis for understanding how EPS can scavenge free radicals enhance immunity, leading to the antitumour and antiaging properties associated with the active groups in the molecular structure, including OH, C=C, C=O, C–O–C, etc.

### Antioxidant activity in vitro

#### DPPH radical scavenging activity

The EPS from *Bacillus subtilis* is an important active substance that could act against various diseases caused by reactive oxygen species (ROS), such as tumours and allergies, which is partly attributed to the capability to scavenge free radicals.

**Figure 4:**
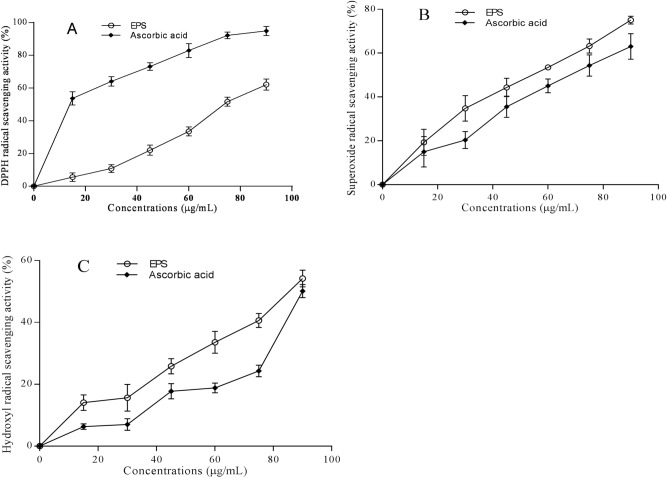
Antioxidant activities of EPS from *Bacillus subtilis* xztubd1 and ascorbic acid against DPPH radicals (**A**), superoxide radicals (**B**), and hydroxyl radicals (**C**). Data are expressed as the mean ± standard error.

As shown in Fig. 4A, EPS and ascorbic acid scavenged DPPH radicals in a concentration-dependent manner. At the same and highest concentration (90 μg/mL), the scavenging rate of EPS was approximately 62%, which is approximately 1.5 times lower than that of ascorbic acid (94.9%). Although the value of EPS is remarkably lower than that of ascorbic acid, EPS exhibited promising scavenging activity.

#### Superoxide radical scavenging activity

The superoxide anion radical scavenging rates of EPS and ascorbic acid at concentrations of 0–90 μg/mL are shown in Fig. 4B. EPS and ascorbic acid again showed concentration-dependent scavenging radical activities. The scavenging rates of EPS were significantly higher than those of ascorbic acid (P < 0.05), as EPS reached 75%, which is approximately 1.2-fold higher than that of ascorbic acid at 90 μg/mL (63%). These results indicated that EPS may be an excellent antioxidant that can be applied in practice.

#### Hydroxyl radical scavenging activity

To further reveal the antioxidant capacity of EPS, the hydroxyl radical scavenging rate was measured. As shown in Fig. 4C, consistent with the data from the DPPH and superoxide radical scavenging activity assays, the hydroxyl radical scavenging rate of EPS increased in a concentration-dependent manner, showing 54% activity at 90 μg/mL, which was markedly higher than that of ascorbic acid. Thus, EPS may become a fine scavenger for oxygen radicals.

Taken together, Fig. 4 shows that the EPS from *Bacillus subtilis* xztubd1 had a higher ability than ascorbic acid to scavenge ROS, as demonstrated by its ability to scavenge the three kinds of radicals mentioned above, to prevent oxidative damage in a dose-dependent manner. Furthermore, the ability of EPS to scavenge superoxide and hydroxyl radicals was significantly higher than that of ascorbic acid, which suggests that EPS may be applied in medicine to treat ROS-induced diseases and inflammation.

### Assessment of EPS immune activity in mice

#### Determination of immune organ indices

**Figure 5:**
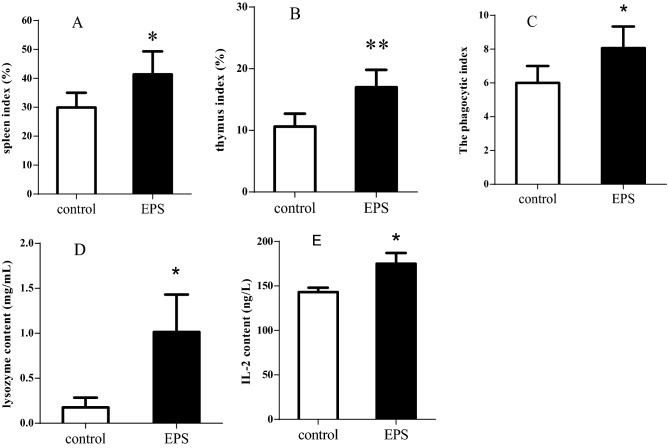
Immune-strengthening activities of EPS from *Bacillus subtilis* xztubd1 on immune organ indices (**A**,**B**), phagocytic activity of macrophages (**C**), lysozyme activity in macrophages (**D**) and IL-2 content in serum (**E**). Data are expressed as the mean ± standard error (n = 6). *P < 0.05, **P < 0.01 versus control.

To systematically investigate the effects of EPS on the immune system in mice, the thymuses and spleens of the two groups of mice were weighed, and the immune organ indices were evaluated (Fig. 5A,B). EPS treatment significantly increased the thymus and spleen indices compared with the control group, which indicated that EPS can boost the functions of immune organs. These results suggest that EPS enhanced immunity and was involved in thymus-mediated cellular immunity and spleen-mediated humoral immunity in mice.

#### Phagocytic activity of macrophages

Macrophages play a critical role in innate immunity, adaptive immunity, anti-inflammation and decreasing immune reactions. To confirm the effects of EPS on the phagocytic activity of macrophages, we examined the phagocytic activity and lysozyme activity of EPS in the two groups of mice. As shown in Fig. 5C, the phagocytic activity of macrophages was much higher in the EPS treatment group than in the control group, which indicated that the potential functions of EPS were related to enhancing nonspecific immune functions in mice.

#### Lysozyme activity in macrophages

To further investigate the effects of EPS on macrophages, lysozyme activity was examined in the control and treatment groups. Compared to the control group, the EPS treatment group displayed a significant increase in lysozyme activity (Fig. 5D). The results showed that EPS notably enhanced the lysozyme activity of macrophages and improved nonspecific immune function.

#### Assessment of IL-2 in mice

IL-2, the first cytokine that is a potential therapeutic target for the modulation of immune responses, boosts the immune response in cancer and autoimmune diseases and promotes the generation of T_reg_ cells, B cell proliferation, the secretion of antibodies and macrophage activation. The expression of IL-2 was clearly elevated in the EPS treatment group compared to the control group (Fig. 5D). The results showed that EPS is associated with reinforcing the immune function of IL-2, which contributes to the treatment of diseases associated with the immune system.

#### Growth suppression effects on HeLa cells caused by EPS

**Figure 6:**
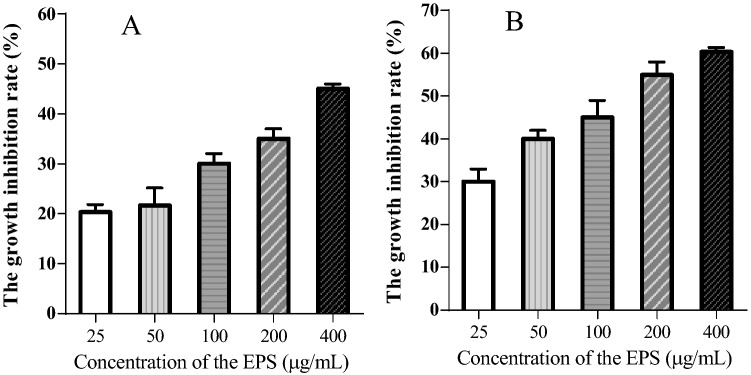
The growth inhibitory effects of EPS on HeLa cells for 24 (**A**) and 48 h (**B**). Data are expressed as the mean ± standard error (n = 6).

Recently, cancer therapy has become increasingly valued, and cancer drug development is a significant part cancer treatment. Because of the functional diversity of microbial metabolites, developing drugs from microorganisms, especially probiotics, is feasible and indispensable. To examine the effects of EPS on cancer in vitro, we examined the inhibition ratio of HeLa cells by MTT assay. The growth suppression ratios caused by EPS on HeLa cells over a range of concentrations (25–400 μg/mL) for 24 and 48 h are shown in Fig. 6. EPS reduced the growth of HeLa cells in a dose- and time-dependent manner. The inhibition ratios increased with increasing EPS concentration and incubation time. At the highest concentration (400 μg/mL), the inhibition ratios reached approximately 45% and 60% at 24 h and 48 h, respectively, which indicated that EPS is toxic and can inhibit cancer cell growth and proliferation in vitro; thus, EPS is expected to be an antitumour drug for adjuvant therapy.

## Discussion

In this study, we identified the novel strain *Bacillus subtilis* xztubd1 after isolation from a housefly by morphological observations and gene sequence analysis, which was found to produce large amounts of an EPS. The characterization and bioactivities of the EPS was further examined, including the active functional groups in the molecular structure and antioxidant, enhancing immunity and anti-tumour activities. These results showed that the EPS, with OH, C=C, C=O C–O–C groups, exhibited appreciable antioxidant activity that could potentially be greater than that of ascorbic acid, enhanced immunity and displayed antitumour activity in vitro. Our results provide important evidence to understand a potential mechanism through which the EPS from *Bacillus subtilis* isolated from a housefly maybe treat disease caused by ROS and low immune function. The EPS could be a potential source for a multifunctional anticancer drug due to its toxicity to cancer cells, enhancement of immunity and ability to relieve ROS-induced inflammation.

Because of their multiple health benefits, probiotics (viable and beneficial microbes) have recently gained wide attention, and their potential to modulate host immune responses and treat diabetes, cancers, and so forth have been explored^22^. Moreover, owing to their ability to produce various bioactive substances, developing new microorganism resources has great research and application value. EPSs are synthesized and secreted by various microbes to store nutrients and protect against outside damage^23^. Few studies have reported the biological activities (antioxidant and antitumour activities) of EPSs from *Bacillus subtilis*, but these studies have rarely been applied in practice due to their low levels and narrow range of activity. *Bacillus subtilis* xztubd1 was first isolated from a housefly, and was found to produce a large amount of an EPS. This EPS from *Bacillus subtilis* xztubd1 (a probiotic) has been generally recognized as safe and beneficial to animals and humans and is worthy of further investigation for its chemical components and biological activities.

It has been considered that molecular functions and mechanisms of action are related to chemical structure. UV–vis and FTIR spectroscopies are techniques that measure the absorption of radiation, which determines particular compositions and structures of substances in a sample^24^. Our results showed that the EPS from *Bacillus subtilis* xztubd1 had active functional groups, including OH, C=C, C=O, C–O–C, etc. These data provided a new theoretical basis for understanding how this EPS scavenged free radicals and enhanced immunity, leading to anti-tumour and anti-aging properties.

Reactive oxygen species (ROS) have been associated with inflammation and are known as important regulators of physiological cell signalling^25^. Overproduction of free radicals could lead to biomolecule damage and is considered a risk factor for diseases such as cancer, ageing, arthritis, and diabetes^26^. Our results showed that EPS exhibited good oxygen free radical scavenging activities against DPPH, superoxide and hydroxyl free radicals in vitro, showing values of 62%, 75% and 54%, respectively. Notably, these last two values were greater than those of ascorbic acid. These findings imply that the EPS from *Bacillus subtilis* is an important active substance against various diseases caused by reactive oxygen species (ROS), such as tumours and allergies, which is partly attributed to its capability to scavenge free radicals.

The immune system is composed of the thymus, lymphoid tissues and bone marrow and protects against diseases by activation of the immune response. Disorders of the immune system result in many diseases, such as inflammation, cancer and autoimmune diseases^27^. We demonstrated that the EPS from *Bacillus subtilis* xztubd1 clearly enhances the immune system. The immune organ indices, phagocytosis, lysozyme activity in macrophages and IL-2 content in mice were all significantly elevated. IL-2 is typically produced by activated T cell and promotes lymphocyte grow, proliferation and differentiation. Because of IL-2 induction and enhancement of cytotoxic activity, it was used extensively in clinical research and tumor treatment. Therefore, the EPS can be regarded to promote immune homeostasis, which can enhance the immune response and protect against diseases.

Recently, cancer therapy has become increasingly valued, and cancer drug development is a significant part of cancer treatment. Because of the functional diversity of microbial metabolites, developing drugs from microorganisms, especially probiotics, is feasible and indispensable. Our results showed that EPS (400 μg/mL) inhibited cancer cell growth in vitro, and the inhibition ratios reached approximately 45% and 60% at 24 h and 48 h, respectively. Additionally, EPS has excellent water solubility and safety. These findings imply that EPS has the potential to be used as a drug against cancer.

In summary, our results suggest that the EPS from *Bacillus subtilis* isolated from a housefly could scavenge ROS, promote immune functions and inhibit cancer cell growth, because the EPS contain a variety of chemical functional groups (such as O–H groups, C=C, C=O, C-O–H and C–O–C bonds) and complex structure. The EPS might cure inflammation and tumour by oxygen radicals scavenging activity, improving immune response and inhibition the growth of cancer cells, which provide the theoretical basics for the development and utilization of intestinal probiotics. Furthermore, The EPS is expected to become a potential multifunctional drug and adjuvant therapy for diseases, such as cancer and inflammation. Further studies are needed to determine the mechanisms related to how EPS can cure the specific disease in vivo.

## Conclusion

In this study, our results revealed the structural characterization and multifunctional biological activities of the EPS from a novel species of *Bacillus subtilis*. The EPS had strong reductive groups (such as OH, C=C, C=O, C–O–C) and complex structure with α-pyranose, therefore exhibited the much higher bioactivity, including antioxidant activities, enhancing the immune response and antitumour activity. Moreover, these results not only speculate the mechanism that probiotics offer benefits to the host, but also provide scientific evidence for the development and utilization of the EPS from *Bacillus subtilis*. The greatest challenges at present include further analysis the exact component and structure of the EPS and further investigate the specific mechanism of EPS treatment. Component and structure of EPS medicines were redesigned to improve therapeutic effect for a single disease.

## Materials and methods

### Bacterial isolation and identification and culture conditions for EPS production

A vigorous housefly was captured in a laboratory in October, and its surface was sterilized with alcohol (75%) for 1 min. After the body was ground with silica by mortar sanding, 1 mL of sterile saline solution (0.9% NaCl w/v) was added in a sterile environment, and 0.1 mL of the resulting homogenate solution was added to the surface of LB solid medium with even coverage to incubate at 37 °C for 24 h. A protuberant and sticky bacterial colony was selected for purification and culture, and then the bacterial strain was identified according to Bergey’s Manual of Systematic Bacteriology and 16S rDNA sequence homology analysis (PCR using universal primers 27F: 5'- AGAGTTGATCCTGGCTCAG -3' and 1492R: 5'- GGTTACCTTGTTACGACTT -3'). Luria Bertani (LB) broth was used for EPS production, and the components were as follows (g/L): beef extract 3, peptone 10, and sodium chloride 5, pH 7.0. Three times, an activated strain was transferred to a 10 L automatic fermenter with a 10% inoculum concentration for large-scale culture at 37 °C and 300 rpm for up to 3 days.

### Extraction and purification of the bacterial EPS

The fermentation liquor was collected and centrifuged at 5000*g* for 15 min. The supernatant was concentrated to 1/10 the volume in a rotary evaporator at 50 °C, mixed with 4 volumes of prechilled ethanol, and then allowed to stand at 4 °C for 24 h. Centrifugation was used to obtain the crude EPS, and a powder was prepared by vacuum freeze-drying. The EPS was then further purified by Sephadex G-75 column chromatography.

### Ultraviolet–visible (UV–vis) and Fourier transform infrared (FTIR) spectroscopy of the EPS

UV–vis spectroscopy of the EPS aqueous solutions (1 mg/mL) were acquired in the wavelength range of 200–800 nm. Five milligrams of dried EPS powder was ground with 200 mg of KBr in an agate mortar and compressed into tablets, which were subjected to FTIR analysis from 500 to 4000 cm^−1^.

### Assessment of the antioxidant activity of the EPS in vitro

#### DPPH radical scavenging activity

Two millilitres of different concentrations of EPS and ascorbic acid (as a positive control) ranging from 15 to 90 μg/mL were added to 2 mL of an ethanolic solution of DPPH (0.2 mM) and then mixed well. After 30 min of incubation at room temperature, the absorbance of the mixture, A_i_, was measured at 525 nm. A_j_ is the absorbance of the mixture containing 2 mL of different concentrations of EPS solutions and 2 mL of deionized water. A_c_ is the absorbance of the mixture consisting of 2 mL of deionized water and 2 mL of an ethanolic solution of DPPH (0.2 mM). DPPH scavenging activity was calculated by the following formula: scavenging activity (%) = [1 – (A_i_—A_j_)/A_c_] × 100%.

#### Superoxide radical scavenging activity

Ten millilitres of different concentrations of EPS (0–100 μg/mL) and ascorbic acid (as a positive control) were made in Tris–HCl buffer solution (pH 8.2) and preheated to 25 °C. Then, 1 mL of pyrogallol (3 mM) was added and the solution was mixed well. The absorbance of each mixture was measured at 325 nm every 30 s for 10 min. The scavenging activity was calculated by the equation: scavenging rate (%) = [(K_0_ − K_1_)/K_0_] × 100%, in which K_0_ and K_1_ represent the rate of pyrogallol oxidation of itself and that after the addition of EPS, respectively.

#### Hydroxyl radical scavenging activity.

The Fenton reaction adopted the H_2_O_2_/Fe^2+^ system and was used for the determination of hydroxyl radical scavenging. Each reaction system consisted of 1 mL of different concentrations of EPS or ascorbic acid (as a positive control), 1 mL of FeSO_4_ (9 mM), 1 mL of salicylic acid ethanolic solution (9 mM), and 1 mL of H_2_O_2_ (9.8 mM) and was incubated at 37 °C for 50 min. The absorbance of each mixture was measured at 510 nm to give the value A_i_. The scavenging activity was expressed by the following formula: scavenging rate (%) = [1- (A_i_—A_j_)/A_c_] × 100%, where A_j_ is the absorbance value of the system that replaced H_2_O_2_ with deionized water, and A_c_ (blank control) is the absorbance of the system that replaced the EPS solution with deionized water.

### Animal treatment protocols

Six-week-old Kunming male mice (weighing 18−20 g) were purchased from the Experimental animal center of the Tumor Hospital of Shanxi (China). The animals were provided abundant food and sterile water and housed in a sterile environment. The mice were randomly assigned to one of the following two groups, each consisting of six mice: control group and experimental group. Mice in the control group had free access to sterile water, whereas the experimental groups drank 1 mL of a sterile water solution containing EPS (up to 200 mg/kg) prior to sterile water. Mice were anaesthetized and sacrificed on Day 16 to be used immediately in the following experiments.

All animal experiments were approved by the Institutional Animal Care and Use Committee of Shanxi University and were conducted in strict accordance with Chinese national standards (GB/T35823-2018).

### Assessment of immune activity of the EPS in mice

#### Determination of immune organ indices

The sacrificed mice were dissected, and their thymus and spleen were removed and weighed with an electronic analytical balance. The immune organ indices were calculated by the following formulas: spleen index = spleen weight (mg)/mouse weight (g); and thymus index = thymus weight (mg)/mouse weight (g).

#### Phagocytic activity of macrophages

On Day 16, each mouse was weighed, and 0.1 mL of ink diluted threefold with normal saline was injected into the tail vein. Then, blood (20 μL) was sampled from the tip of the mouse tail at 2 min and 10 min. The blood samples were immediately added to 2 mL of Na_2_CO_3_ solution (0.1%) and mixed well. The absorbance of each mixture was measured at 675 nm, and Na_2_CO_3_ solution (0.1%) served as a blank control. The livers and spleens of the mice were collected and weighed. The phagocytic index (α) was calculated by the following formula: α = [body mass/(liver mass + spleen mass)] ×  $\root 3 \of K$; $K = \frac{{\lg {A_1} - \lg {A_2}}}{{{t_2} - {t_1}}}$; where A_1_ and A_2_ are the absorbance of the blood sample at 2 min and 10 min, respectively, and t_1_ and t_2_ are 2 min and 10 min, respectively.

#### Lysozyme activity in macrophages

The mice were sacrificed by cervical dislocation, and then the peritoneal macrophages were rinsed with 1 mL of normal saline. The lysozyme contents were measured using assay kits according to the manufacturer’s instructions (Nanjing, China).

#### Assessment of interleukin-2 (IL-2) in mice

Blood samples were collected from the mice and centrifuged to obtain serum. Serum IL-2 levels were detected by ELISA using assay kits according to the manufacturer’s instructions (Nanjing, China).

#### The suppression rate of HeLa cells

Antitumour activity was measured by MTT assay as described^26^. Briefly, after HeLa cells (obtained from National Collection of Authenticated Cell Cultures China) were cultured in RPMI-1640 in 96-well microtiter plates (2.0 × 10^4^ cells per well) under 5% CO_2_ at 37 °C for 12 h, the cells were treated with different concentrations of EPS (25, 50, 100, 200 or 400 μg/mL) for 24 h or 48 h, with each sample concentration having 6 replicates. The culture fluid was discarded, and the cells in each well were rinsed three times with PBS. Then, 200 μL of fresh medium and 20 μL of MTT reagent (5.0 μg/ml) was added to each well. After the HeLa cells were cultured for an additional 4 h and the medium was removed, 150 μL of dimethyl sulfoxide (DMSO) was added to each well and the plate shaken well on a horizontal shaker for 10 min. The absorbance of each well was then measured with a microplate reader at 530 nm. The inhibition ratio was calculated as follows: inhibition ratio (%) = [1 − (A_sample_/A_control_)] × 100.

### Statistical analysis

Statistical analysis, including one-way analysis of variance and significance testing, was conducted using IBM SPSS Statistics 20 (IBM, Armonk, NY, USA). All experimental data are expressed as the mean ± standard error. Statistical significance was defined as p < 0.05.

### Ethical approval

All animal experiments were approved by the Institutional Animal Care and Use Committee of Shanxi University and were conducted in strict accordance with Chinese national standards (GB/T35823-2018).

### Consent for publication

All authors agreed to the publication of the data reported in this work.

## Data Availability

We confirm the study is conducted in accordance with ARRIVE guidelines (https://arriveguidelines.org).

## Abbreviations

EPS: Extracellular polymeric substance
ROS: Reactive oxygen species
UV–vis spectroscopy: Ultraviolet–visible spectroscopy
FTIR spectroscopy: Fourier transform infrared spectroscopy
IL-2: Interleukin-2
PCR: Polymerase chain reaction
DPPH: 1, 1-Diphenyl-2- picrylhydrazyl
DMSO: Dimethyl sulfoxide
MTT: Methyl thiazolyl tetrazolium
LB broth: Luria Bertani broth
